# Ecological Risk Assessment of Heavy Metals in Water Bodies around Typical Copper Mines in China

**DOI:** 10.3390/ijerph17124315

**Published:** 2020-06-17

**Authors:** Jingchao Liu, Jin Wu, Weiying Feng, Xia Li

**Affiliations:** 1 College of Architecture and Civil Engineering, Beijing University of Technology, Beijing 100124, China; liujingchao721@126.com; 2 School of Space and Environment, Beihang University, Beijing 100191, China; fengweiying@buaa.edu.cn; 3 Beijing Advanced Innovation Center for Big Data-Based Precision Medicine, Beihang University, Beijing 100191, China; 4 College of Water Science, Beijing Normal University, Beijing 100875, China; 5 China Geological Environment Monitoring Institute, Beijing 100081, China

**Keywords:** copper mine, heavy metal, ecological risk, species sensitivity distribution

## Abstract

In order to understand the heavy metal pollution status and ecological effect in aquatic environment around copper mine areas, seven heavy metals (Cd, Cd, Cr, Cu, Hg, Zn, the Ni, and Pb) in aquatic environments in seven representative copper mine regions were selected from the literature in 2005–2013 for ecological risk assessment by using potential ecological risk index, geoaccumulation index, nemerow index and species sensitivity distribution method (Potential Affected Fraction (PAF) and Multi-Substance PAF (MSPAF)). The results of sediment ecological risk analysis showed that Cd, Cu and Pb were the main pollutants in sediments. The results of species sensitivity distribution analysis showed that the HC5 values (Hazardous Concentration for 5% of species) of seven heavy metals were different with order Zn > Cr > Cd > Pb > Cu > Ni > Hg. The MSPAF of seven copper mines in the following order with species sensitivity distribution method was as follows: Dabaoshan (99%) = Dahongshan (99%) = Baiyin (99%) > Dexing (97%) > Jinchuan (92%) > Tongling (39%) > Daye (24%). This study analyzes the impact of copper mining on the aquatic environment, and the results of this study will be great value for the comprehensive pollution governance of mining.

## 1. Introduction

In recent years, with the improvement of China’s industrial level, the development of the mining industry has accelerated. A large amount of solid waste and wastewater from mining has become the main cause of water pollution around the mine [1,2,3]. The main components of these solid wastes and wastewater are heavy metals. If these solid wastes and waste liquids are discharged directly into the river without effective recovery and treatment, a large number of harmful heavy metals will enter the river ecosystem directly in the form of solid or liquid, resulting in serious heavy metal pollution in the river [4,5]. Harmful heavy metals in wastewater migrate and transform among water bodies, sediments, and organisms, including adsorption, parsing, precipitation, and biological absorption [6,7]. Generally speaking, heavy metals in the water can pose a direct or indirect threat to the ecosystem through biological enrichment and amplification, eventually affecting human health by passing through the food chain step by step [8]. Compared with river water, heavy metals in sediments are characterized by high abundance, easy enrichment and easy detection, which are indicators of river pollution and can effectively reflect the pollution of water [9,10]. In addition, sediment records of various pollutants in the industrial civilization origin, distribution, migration and transformation history reflect human activity, natural environment interactions, and the transition and enrichment of heavy metals in the river ecosystem. Such environmental geochemical information is critical for the evaluation of environment status and trend, and the detection of river trace heavy metal pollution history [11,12].

Environmental pollution and the treatment of mine water, evolution of water environment and utilization of water resources have always been the focus of integrated studies on mine resources and environment. In particular, a large number of studies have been carried out on the characteristics of heavy metal pollution in the mine environment [13,14,15]. However, there are relatively few studies on the toxicological effects of the mine ecological environment. With the complexity of environmental issues and the increasing demand for water security, the study on mine water environment has gradually shifted from simple pollution to environmental and ecological risks [16,17,18]. From the perspective of risk management and control, the wastewater from mining not only harms the surrounding water environment, but also poses systemic risks to the ecological structure and human health [19].

China is currently one of the major producers, consumers and exporters of many heavy metals including copper. A large number of studies have shown that solid waste and wastewater from copper mining have posed serious harm to the river ecosystem [20,21,22]. For example, Li et al. [23] studied the heavy metal morphology and its potential ecological risks in the sediments of Huixi River. Teng et al. [24] discussed the pollution assessment benchmark of heavy metals in river sediments around Dexing copper mine. In general, there is still a lack of systematic research on the ecological effects of the water environment in copper mining in China.

Therefore, this paper selected typical copper mines in China as research objects, and studied the distribution characteristics, ecological risks and species sensitivity distribution of seven heavy metal elements including Cd, Cu, Zn, Hg, Pb, Cr and Ni in water and sediments. This study provides (1) a theoretical and scientific basis for the heavy metal pollution control project in the surrounding water bodies of the mine, and (2) support for the ecological risk management of the surrounding water bodies of the mine.

## 2. Materials and Methods

### 2.1. Data Source and Processing

**Figure 1:**
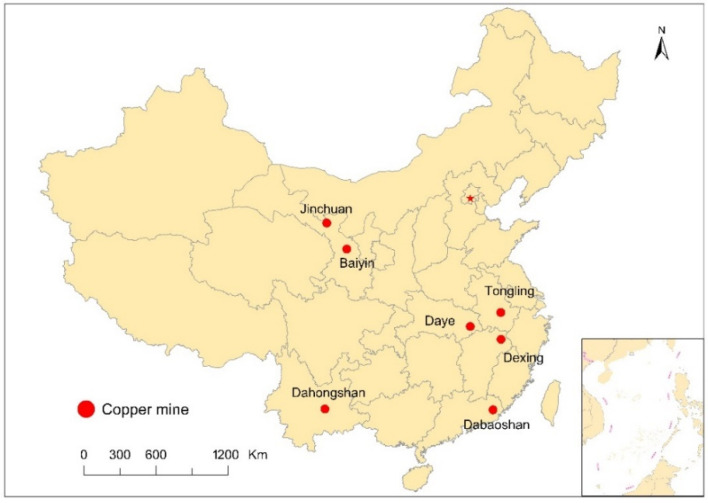
Location of seven representative copper mines in China.

The study covers seven copper mines located in six provinces in China. Seven heavy metals (Cd, Cu, Zn, Hg, Pb, Cr and Ni) content data in river water sediments were collected from the literature in 2005–2013 from three major online literature databases (Web of Science, Science Direct and China National Knowledge Infrastructure). The criteria for collecting the scientific literatures were: (1) China’s top 20 copper mines, (2) the literature, and (3) the highest possible regional distribution. Therefore, the selected copper mines are the Tongling [25], Dabaoshan [26,27], Dahongshan [28], Daye [29], Jinchuan [30], Baiyin [30], and Dexing copper mines [31]. Figure 1 shows the specific distribution location of each copper mine.

The methods for the determination of heavy metals in the literature are the standard methods recommended by the state or revised according to the standards. It should also be noted that sampling times (publication year) spanned from 2005–2013 and analytical methods are not completely consistent. Therefore, uncertainty is inevitable in pollution and risk assessment. However, content data should be of primary importance among influence factors and further research should focus on comparisons of mine geochemical surveys with similar sampling times. Table 1 shows the methods used in the seven studies to determine the concentration of heavy metals.

Heavy metal elements related to freshwater animal toxicology data come from the USA. Environmental Protection Agency (EPA) website [32], select fresh water medium exposure time less than 10 days of median lethal concentration (LC50) or half maximal effect concentrations (EC50) toxicology data [33]. Species include algae, fungi, amphibians, crustaceans, fish, insects, invertebrates, mollusks, and worms. In order to analyze the effects of different heavy metals on all freshwater organisms, the toxicological data were not grouped by species.

### 2.2. Potential Ecological Risk Index

The potential ecological risk index (RI) method was proposed by Lars Hakanson in the 1980s as a classic method to evaluate the potential ecological risk of heavy metals in sediments [34]:

(1) $$C_{f}^{i}=\frac{C_{D}^{i}}{C_{R}^{i}}$$

(2) $$E_{r}^{i}=T_{r}^{i}\times C_{f}^{i}$$

(3) $$\mathrm{RI}=\overset{n}{\underset{i=1}{\sum }}E_{r}^{i}$$

where $C_{f}^{i}$ the pollution index for a given heavy metal, $C_{R}^{i}$ is the reference value of heavy metal in the sediment, $C_{D}^{i}$ is the present concentration of heavy metal, $E_{r}^{i}$ is heavy metal potential ecological risk factor, $T_{r}^{i}$ is the toxic response factor for a single heavy metal contamination, and RI is the total potential ecological risk index for heavy metals. The reference values of sediment background values were the Chinese soil background values. The background values of Cd, Cu, Pb, Cr, Zn, Hg and Ni were 0.07, 20, 23.6, 53.9, 67.7, 0.04 and 24.9 mg/kg, respectively. When RI < 150, the risk level is low; when 150 ≤ RI < 300, the risk level is medium; when 300 ≤ RI < 600, the risk level is high; and when RI ≥ 600, the risk level is very high [34].

### 2.3. Geoaccumulation Index

The geoaccumulation index ($I_{\mathrm{geo}}$) method is a method often used to evaluate the pollution level of heavy metals in sediments. This method can effectively eliminate the influence of natural geological accumulation [35,36]:

(4) $$I_{\mathrm{geo}}=\log_{2}\left(\frac{C_{n}}{1.5\times B_{n}}\right)$$

where $C_{n}$ is the measured concentration, $B_{n}$ is the sediment background reference value, and 1.5 is the background matrix correction coefficient. $I_{\mathrm{geo}}$ < 0 is practically unpolluted; 0 < $I_{\mathrm{geo}}$ < 1 is unpolluted to medium polluted; 1 < $I_{\mathrm{geo}}$ < 2 is medium pollution; 2 < $I_{\mathrm{geo}}$ < 3 is medium pollution to heavy pollution; 3 < $I_{\mathrm{geo}}$ < 4 is heavy pollution; 4 < $I_{\mathrm{geo}}$ < 5 is heavy pollution to extremely heavy pollution;${\;I}_{\mathrm{geo}}$ > 5 is extremely heavy pollution.

### 2.4. Nemerow Index

The nemerow index (>*P_N_*) can take into account the contents of all heavy metals and make a comprehensive evaluation of the pollution level of heavy metals in sediments [36,37]:

(5) $$P_{N}=\sqrt{\frac{AvgP_{i}^{2}+MaxP_{i}^{2}}{2}\;}$$

where $P_{i}$ is a single pollution index, $P{\;}_{i}$=$\;C_{i}$/$S_{i}$. $C_{i}$ is the measured concentration and $S_{i}$ is the pollutant concentration standard value. *Max*$P_{i}$ and *Avg*$P_{i}$ are the maximum and average values of all index $P_{i}$, respectively. The method divides pollution into five levels: *P_N_* ≤ 0.7, safety domain; 0.7 < *P_N_* ≤ 1.0, precaution domain; 1.0 < *P_N_* ≤ 2.0, slightly polluted domain; 2.0 < *P_N_* ≤ 3.0, moderately polluted domain; *P_N_* > 3, seriously polluted domain.

### 2.5. Species Sensitivity Distribution

The species sensitivity distribution (SSD) selects the Burr type Ⅲ distribution of cumulative exposure concentration curves fitting the Burr type Ⅲ distribution of skewness and kurtosis; in covering the larger range, it can make it more flexible fitting the parameters [38,39,40]. The Burr type Ⅲ distribution fits the parameters of the function equation for:

(6) $$F\left(x\right)=\frac{1}{{\left[1+{\left(\frac{b}{x}\right)}^{c}\right]}^{k}}$$

HC5 is the cumulative concentration on the species sensitivity distribution curve corresponding to the damage to 5% of species. The smaller the hazardous concentration for 5% (HC5), the more toxic the heavy metals represented [41,42]. The formula for calculating HC5 by using Burr type Ⅲ distribution is:

(7) $$HC\left(q\right)=\frac{b}{{\left[{\left(\frac{1}{q}\right)}^{\frac{1}{k}}-1\right]}^{1/c}}$$

where *q* is the corresponding protection level. potentially affected fraction (PAF) is the proportion of hazardous species corresponding to the measured concentrations on the species sensitivity distribution curve, and the calculation formula is as follows:

(8) $$PAF\left(x\right)=\frac{1}{{\left[1+{\left(\frac{b}{x}\right)}^{c}\right]}^{k}}$$

where *x* is the measured concentration of pollutant. multi-substance potentially affected fraction (MSPAF) is the proportion of hazardous species caused by multiple pollutants, which can reflect the combined pollution caused by multiple pollutants in water. The calculation formula is:

(9) $$MSPAF=1-\overset{n}{\underset{i=1}{\prod }}\left(1-PAF_{i}\right)$$

## 3. Results

### 3.1. Heavy Metals in River Water

The distribution of heavy metal concentration in water around typical mines in China is shown in Table 2. It can be seen from the table that the contents of various heavy metals vary greatly. The average concentrations of Zn, Cu and Pb are 9.032, 7.656 and 1.655 mg/L, respectively, which are the main heavy metal elements in the water around the copper mine. The average concentration of Hg is 0.0001 mg/L, the lowest among the seven heavy metals. The average concentrations of Cd, Cu, Zn, Pb, Cr and Ni in the surrounding rivers of Dahongshan, Baiyin and Dabaoshan copper mines all exceeded the World Health Organization Drinking Water Standard (WHO). In addition to Hg, the concentrations of heavy metals in the surrounding waters of seven typical copper mines in China all exceeded the standard to varying degrees. The excessive concentration of these seven heavy metals is closely related to the development of mining activities. There are many ways in which mining wastewater can contaminate natural water bodies. When Wang et al. [43] studied the groundwater of Dahongshan, they found that the heavy metals in the groundwater would enter the river water through the way of lateral recharge of the river. Gou et al. [44] found that the acid mine wastewater in the 4# tailings pond would release the heavy metals in the tailings and pollute the water resources.

### 3.2. Heavy Metal in Sediment

The distribution of heavy metals in the sediments around copper mines is shown in Table 3. The concentration of each heavy metal is Cr > Cd > Cu > Pb > Zn > Hg > Ni in descending order, and the distribution of heavy metals in sediments is similar to that in water. Heavy metal pollution in river bottom sediments is mainly due to the deposition of heavy metals in water and the adsorption of heavy metals by riverbed sediment [46,47]. It can be seen from the table that the heavy metal pollution in sediments in different regions is quite different. Among them, the total heavy metal content in Jinchuan sediment is up to 19,057.53 mg/kg, in which the mean contents of Cu and Ni are 7265.37 and 7781.03 mg/kg respectively, which are 350 and 300 times the background value, while the total heavy metal content in the water sediment around Dahongshan is 228.8 mg/kg, only Cu and Ni exceed the background value. Yan et al. [48] found that the concentration of Zn, Cu and Pb in sediments in a typical copper mining area in southern China was significantly higher than that of other metals. The difference of distribution of heavy metals in the sediment of copper mine rivers may be related to the discharge of mine wastewater, the accumulation of mining tailings in the river, the sand mining and the river function. Although river sediments can absorb some heavy metals to reduce water pollution, they will also release heavy metals to water, causing secondary pollution that is difficult to control [49,50].

### 3.3. Sediment Ecological Risk Analysis

According to the evaluation of the heavy metal pollution degree of the sediments in the surrounding rivers of seven mining areas by the potential ecological risk index, it can be seen from Table 4 that the ecological risk of a single heavy metal in the sediments is in the order of Cd > Cu > Pb > Ni > Hg > Zn > Cr from large to small. It can be seen that Cd is the main source of potential ecological risks in the sediments surrounding the copper mine, and Cd has far more influence on the water ecology around copper mines than other metals. The potential ecological risk index of each copper mine was Jinchuan, Baiyin, Tongling, Daye, Dabaoshan, Dexing and Dahongshan in the order of large to small. Among them, Jinchuan, Baiyin, Tongling, Daye and Dabaoshan all achieved extremely high ecological risks, while Dahongshan had relatively low ecological risks. It can be seen that ecological risks have been prevalent in the rivers around the copper mines.

**Figure 2:**
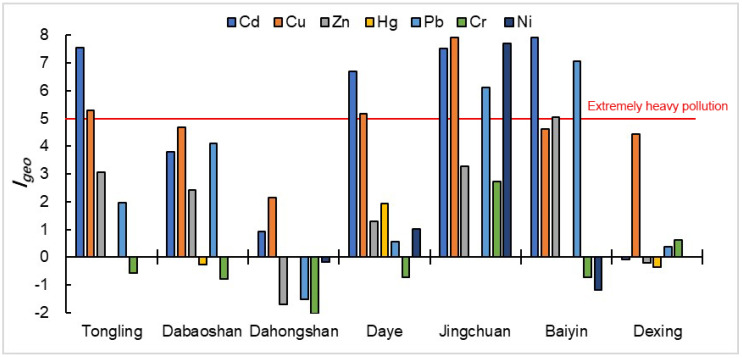
Geoaccumulation index of sediments in rivers surrounding typical copper mines.

**Figure 3:**
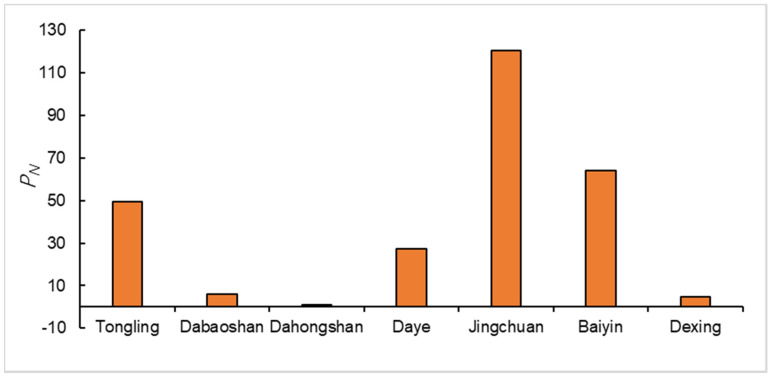
Nemerow index of sediments in rivers surrounding typical copper mines.

Through the assessment results (Figure 2) of the geoaccumulation index method, we can find that the seven heavy metals $I_{\mathrm{geo}}$ values vary widely in different regions. First of all, we can find that Cd, Cu and Pb are the main ecological risk sources among the seven heavy metals. In addition, the pollution status of Cr pollution in most areas is relatively minor, and only the Jinchuan copper mine is in moderate pollution. Due to the pollution caused by the accumulation of Cd, Cu and Pb in sediments, the analysis results of the nemero index (Figure 3) also show that other areas except Dahongshanare in the field of serious pollution. At the same time, it is not difficult to find that excessive accumulation of Cd, Cu and Pb in sediments is closely related to copper mining. The discharge of mine wastewater results in the deposition of a large number of heavy metal solid particles in the river channel. In addition, the solute form of heavy metals in the water will also be adsorbed by the sediment. Heavy metals in sediments can seriously harm the living environment of benthic organisms and cause heavy metals to accumulate in benthic organisms, thus harming the ecological environment.

### 3.4. Species Sensitivity Analysis Based Ecological Risk Assessment

**Figure 4:**
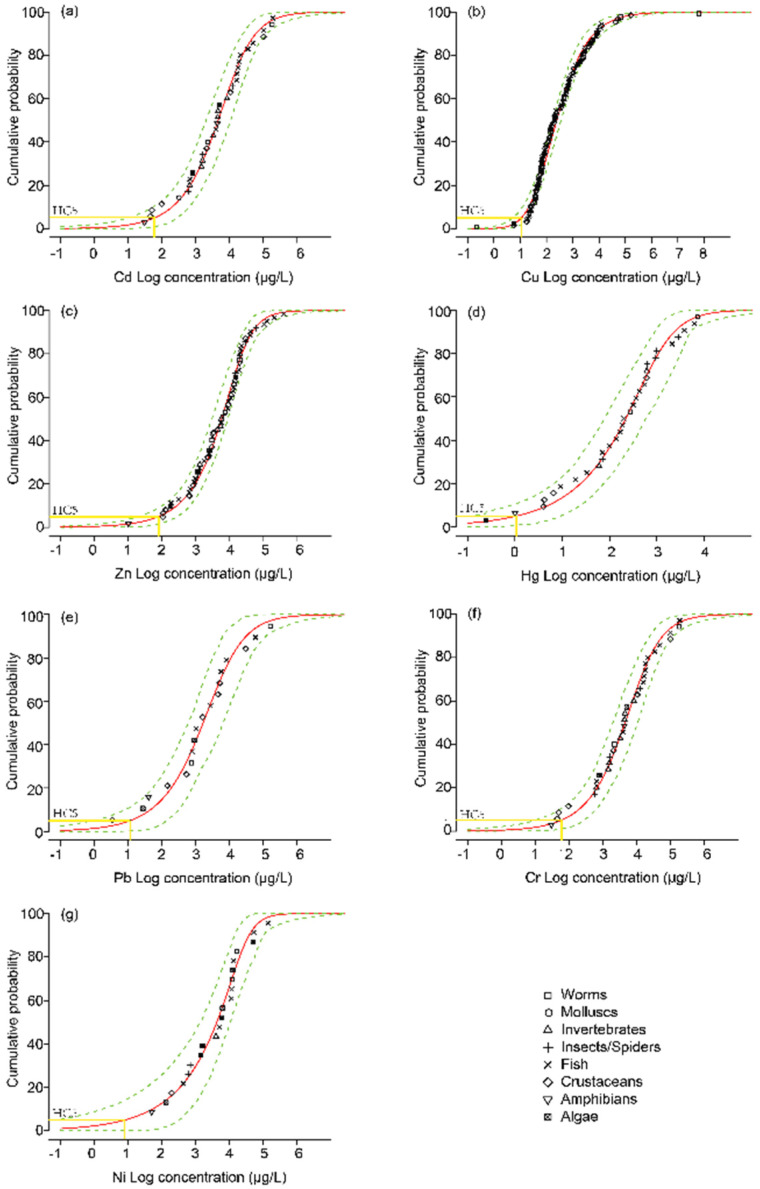
Species sensitivity distribution curves of seven heavy metals to all species. (**a**)-SSD curve for Cd; (**b**)-SSD curve for Cu; (**c**)-SSD curve for Zn; (**d**)-SSD curve for Hg; (**e**)-SSD curve for Pb; (**f**)-SSD curve for Cr; (**g**)-SSD curve for Ni.

Figure 4 shows the distribution curve of species sensitivity using BurrlizO software (Commonwealth Science and Industrial Research Organisation, Canberra, Australia,) launched by the Australian Commonwealth Scientific and Industrial Research Organization CSIRO. Different heavy metals have different HC5, PAF and MSPAF values and the HC5 values of seven heavy metals ranged from large to small as Zn>, Cr>, Cd, >, Pb, >, Cu, >, Ni, >Hg. With the increase of HC5 value, the ecological risk of corresponding heavy metals decreased gradually. Therefore, the proportion of species harmed by Hg was the largest, while that of species harmed by Zn was the smallest. The HC5 values of Cd, Cu, Hg, Ni and Pb are all less than 20 g/L, which are the main source of the ecological risks to water, while the HC5 values of Zn and Cr are relatively large but have a relatively small impact on water ecology. According to the results of the species sensitivity distribution curve, Cu is the main ecological risk source in the water bodies around the nine copper mining areas, and the PAF of Cu in the water bodies around the Dabaoshan, Dabongshan, Jinchuan, Baiyin and Dexing regions all exceed 80%, due to the discharge of copper mine wastewater and the accumulation of copper-bearing tailing sands at the bottom of the riverbed. The PAF of Cd and Cr in the surrounding waters of the copper mine is basically less than 10%, and the species sensitivity is relatively low. Only the PAF of Cd in silver and the surrounding waters of Dahongshan is more than 44%, which is a serious potential ecological risk. The ecological risks of the seven heavy metals in the surrounding waters of Morocco are relatively small, with PAF levels not exceeding 10%.

As can be seen from Table 5, MSPAF of compound ecological risks in water bodies around the 9 mining areas is listed here from the largest to the smallest: Dabaoshan (99%) = Dabongshan (99%) = Baiyin (99%) > Dexing (97%) > Jinchuan (92%) > Tongling (39%) > Daye (24%). Dabaoshan and Dahongshan compounds’ ecological risk has reached 99%, Dexing and Jinchuan compounds’ ecological risk is also more than 80%, the most visible copper mines surrounding water produced by heavy metal compound ecological risk has reached a serious level, causing serious environmental problems for freshwater creatures. Although the community structure of species in different regions is different, the compound ecological risk still has guiding value. Cu, Zn and Pb are the main causes of ecological risks in the surrounding water bodies of typical copper mines. The increase of these heavy metals in the water body is closely related to the discharge of mine wastewater, the accumulation of tailing sand in river channels and the leaching of heavy metals in soil and other copper mining activities.

### 3.5. Limitations

There are 2159 copper mining areas in China, and 18 10,000-ton copper mining areas. The selected copper mining areas may not represent the pollution status of the surrounding water bodies of copper mines in China. In addition, due to the different studies on the acquired data, the obtained data may have certain differences, affecting the consistency of the results. But the methods used in these studies are similar and widely accepted by the academic community, so these differences do not affect the credibility of the results. The biotoxicology data were selected from the EPA database in the United States, but there were some differences between the species in each mine and the selected species, which increased the uncertainty of ecological risk analysis.

## 4. Conclusions

In this study, we analyzed the spatial distribution characteristics and ecological risk levels of seven heavy metals, including Cd, Cu, Zn, Hg, Pb, Cr and Ni, in the water and sediments around seven typical copper mines in China. The results show that the water and sediments around copper mountain generally contain a large amount of heavy metal elements, and the values are much higher than the regional background values. It should also be noted that the sampling time (publication year) spanned from 2005 to 2013 and analytical methods are not completely consistent. Therefore, uncertainty is inevitable in pollution and risk assessment. However, content data should be of primary importance among influence factors and further research should focus on comparison of mine geochemical survey with similar sampling time.

It can be seen from the potential ecological risk index and geoaccumulation index that Cu, Pb and Cd in the sediments are the main ecological risk sources. It can be seen from SSD analysis that Cu, Pb and Zn are the main ecological risk sources in water. At the same time, it is not difficult to find that the surrounding water environment of the copper mine has been seriously polluted by mining activities; the survival of all kinds of creatures in the water has also been seriously threatened, and the river ecosystem has been damaged to a certain extent. In addition, the water pollution will seriously threaten the water safety of the residents around the copper mine, thus causing all kinds of health and safety problems. The evaluation data of this study indicate that the mine will have a huge impact on the surrounding water body, and it is a region that the government should focus on when carrying out ecological risk management

## Figures and Tables

**Table 1:** Analysis methods of heavy metal elements used in seven papers.

Copper Mine	Sediment		Water	
	Heavy Metal	Analysis Methods	Heavy Metal	Analysis Methods
Tongling [25]	Cu, Zn, Pb, Cr	XRF	Cd, Cu, Zn, Cr	AFS
	Cd	AAS	Hg	XRF
Dabaoshan [26,27]	Cd, Cr	ICP-MS	Cd, Cu, Zn, Pb	ICP-OES
	Hg, Pb	AFS		
	Zn, Cu	ICP-OES		
Dahongshan [28]	Cu, Cr, Cd, Pb, Zn, Ni	ICP-MS	Cu, Cr, Cd, Pb, Zn, Ni	ICP-MS
Daye [29]	Cd, Cr, Ni	ICP-MS	Cd, Cr, Ni	ICP-MS
	Cu, Zn,	ICP-OES	Cu, Zn,	ICP-OES
	Hg, Pb	AFS	Hg, Pb	AFS
Jinchuan [30]	Cu, Zn, Pb, Cr, Cd, Ni	ICP-AES	Cu, Zn, Pb, Cr, Cd, Ni	ICP-AES
Baiyin [30]	Cu, Zn, Pb, Cr, Cd, Ni	ICP-AES	Cu, Zn, Pb, Cr, Cd, Ni	ICP-AES
Dexing [31]	Cu, Zn, Cr	ICP-AES	Cu, Zn, Cr	ICP-AES
	Cd, Hg	AAS	Cd, Hg	AAS

XRF—X-ray Fluorescence Spectrometer; AAS—Atomic Absorption Spectroscopy; ICP-MS—inductively coupled plasma massspectrometry; AFS—Atomic Fluorescence Spectrometry; ICP-OES—Inductively Coupled Plasma Optical Emission Spectrometer; ICP-MS—inductively coupled plasma massspectrometry.

**Table 2:** Distribution characteristics of heavy metal concentration in water around typical copper mines (mg/L).

Region	Cd	Cu	Zn	Hg	Pb	Cr	Ni
Tongling [25]	0.0001–0.083	0.001–0.374	0.001–0.72	0.000011–0.000058	-	0.002–0.012	-
	(0.014)	(0.083)	(0.129)	(0.000043)	-	(0.0058)	-
Dabaoshan [26]	0.002–0.1	0.008–13.82	0.021–50.83	-	0.001–2.91	-	-
	(0.031)	(3.92)	(14.35)	-	(8.08)	-	-
Dahongshan [28]	0.004–0.03	0.39–12.7	33.9–57	-	0.3–0.56	9.37–14.9	2.23–3.51
	(0.016)	(9.14)	(42.86)	-	(0.4)	(11.78)	(2.96)
Daye [29]	0.00001–0.125	0.00096–0.019	0.014–0.32	0.0002–0.0003	0.00003–0.00032	0.00002–0.016	0.0011–0.048
	(0.024)	(0.0087)	(0.073)	(0.00023)	(0.00011)	(0.0028)	(0.02)
Jingchuan [30]	0.001–0.005	0.018–22.41	0.007–0.14	-	0.01–0.14	0.003–0.983	0.057–6.85
	(0.001)	(3.086)	(0.0059)	-	(0.054)	(0.167)	(1.427)
Baiyin [30]	0.83–1.82	3.581–40.32	1.69–7.69	-	0.048–2.551	-	-
	(1.18)	(13.82)	(3.71)	-	(1.36)	-	-
Dexing [31]	0.00004–0.054	0.00003–135.99	0.00003–11.18	0.000009–0.00008	0.00011–0.25	0.0047–0.64	-
	(0.0082)	(23.53)	(2.04)	(0.000047)	(0.024)	(0.14)	-
WHO [45]	0.003	2	0.05	0.001	0.01	0.05	0.02

-: not available.

**Table 3:** Distribution characteristics of heavy metal concentration in sediments of typical copper deposits in surrounding rivers (mg/kg).

Region	Cd	Cu	Zn	Hg	Pb	Cr	Ni
Tongling [25]	1.18–79.55	374–2181.8	198–2162.5	-	53.9–410	30.6–132.7	-
	(19.67)	(1167.13)	(844.89)	-	(137.81)	(54.23)	-
Dabaoshan [27]	0.07–6.2	24–3180	3.4–2780	0.04–0.09	24.9–6400	7.8–154	-
	(1.45)	(766.22)	(545.36)	(0.05)	(606.65)	(47.03)	-
Dahongshan [28]	0.12–0.32	37.86–222.68	21.68–47.91	-	5.76–17.56	15.26–22.68	23.2–47.57
	(0.20)	(132.19)	(31.27)	-	(12.30)	(19.61)	(33.23)
Daye [29]	0.22–41.4	19–1410	199–318	0.12–0.33	19-156	22.1–130	29.4–99.2
	(10.78)	(1074.83)	(246.50)	(0.23)	(52.33)	(49.37)	(75.90)
Jingchuan [30]	12.42–27.37	5317.34–8377	774.3–1264.83	-	752–5507.18	408.8–648.48	5068–13112.
	(19.21)	(7265.37)	(977.27)	-	(2475.33)	(539.32)	(7781.03)
Baiyin [30]	1.17–70.64	79.78–1519.5	117.32–10725	-	143.93–9999	34.63–61.63	9.79–23.91
	(25.31)	(742.24)	(3363.43)	-	(4722.14)	(49.4)	(16.52)
Dexing [31]	0.051–0.18	126–2978	20.6–145	0.015–0.12	27.4–76.1	75.7–233	
	(0.1)	(645.63)	(88.2)	(0.047)	(46.19)	(125.14)	

-: not available.

**Table 4:** Potential ecological risk index of sediments in rivers surrounding typical copper mines.

Region	Cd	Cu	Zn	Hg	Pb	Cr	Ni	RI
Tongling	8430	291.78	12.48		29.19	2		8765.47
Dabaoshan	621.43	191.56	8.05	37.5	128.53	1.75		988.81
Dahongshan	85.71	33.05	0.46		2.61	0.73	6.67	129.23
Daye	4620	268.71	3.64	172.5	11.09	1.84	15.24	5093
Jingchuan	8232.85	1816.34	14.43		524.43	20.01	1562.46	2170.54
Baiyin	10,847.14	185.56	49.68		1000.45	1.83	3.32	12,087.99
Dexing	42.86	161.41	1.30	35.25	9.79	4.64		255.25

**Table 5:** Potential hazard ratio (PAF) and composite hazard ratio (MSPAF) of each heavy metal.

Region	Cd	Cu	Zn	Hg	Pb	Cr	Ni	MSPAF
Tongling	5	30	7			1		0.39
Dabaoshan	8	87	71		76			0.99
Dahongshan	6	91.7	89		29	27	44	0.99
Daye	7	4	5	3	0	1	7	0.24
Jingchuan	1	85	4		11	9	34	0.92
Baiyin	49	93.5	41		47			0.99
Dexing	4	95.2	30	1	7	8		0.97
